# The impact of swimming coaches’ authentic leadership coaching on trust, self-efficacy, service quality, and relationship continuity intention

**DOI:** 10.3389/fpsyg.2025.1640823

**Published:** 2025-10-31

**Authors:** Buom Kim

**Affiliations:** Department of Physical Education, University of Kyungdong, Gyeonggi-do, Republic of Korea

**Keywords:** authentic leadership, coach trust, self-efficacy, service quality, relationship continuity intention

## Abstract

**Purpose:**

This study investigates how swimming coaches’ authentic leadership influences trust, self-efficacy, service quality, and relationship continuity intention among adult recreational swimmers in South Korea. Using structural equation modeling (SEM), it aims to provide insights for enhancing leadership practices in community sports.

**Methods:**

A total of 280 adult recreational swimmers participated in the survey, using validated scales on a 5-point Likert scale. Structural equation modeling and bootstrapping with 5,000 resamples were employed to analyze the relationships among authentic leadership, trust, self-efficacy, service quality, and relationship continuity intention.

**Results:**

The SEM analysis demonstrated excellent model fit (*χ*^2^/df = 2.41, CFI = 0.95, TLI = 0.94, RMSEA = 0.059). Authentic leadership significantly influenced coach trust, which in turn positively affected self-efficacy and service quality. While Hypothesis 4 (coach trust → relationship continuity intention) was not supported, mediation analysis showed that self-efficacy and service quality significantly mediated the relationship, indicating that trust indirectly sustains participants’ continuity intention.

**Conclusion:**

Authentic leadership plays a vital role in fostering trust, self-efficacy, and service quality in community sports programs. These factors indirectly support participants’ sustained engagement, highlighting the importance of leadership development in enhancing the effectiveness of community-based swimming programs.

## Introduction

Participation in sports and physical activity has become a key component of modern wellbeing, offering recognized physical, psychological, and social benefits. In South Korea, data from the Ministry of Culture, Sports, and Tourism (2022) indicate that 61.2% of the population engaged in physical activity at least once a week, with swimming ranked among the most popular and desired activities. Despite its popularity, the long-term engagement of participants in recreational swimming programs remains a challenge, with previous research noting high dropout rates in community-based sports programs, including swimming, often exceeding 30% (Howat and Assaker, 2013; Eime et al., 2016). This concern underscores the need for greater attention to coaching effectiveness and leadership style as critical determinants of participant retention.

Traditionally, sports coaching has emphasized technical instruction and physical training. However, contemporary approaches increasingly highlight the psychological and interpersonal dimensions of coaching, with leadership models such as transformational leadership and athlete-centered coaching receiving growing attention (Lorimer and Jowett, 2014; Vella et al., 2011). Among these, authentic leadership has emerged as a particularly influential framework (Walumbwa et al., 2011a; Soto Garcia et al., 2021). It emphasizes ethical behavior, self-awareness, transparency, and a strong moral compass (Avolio and Gardner, 2005; Walumbwa et al., 2008). Unlike transformational leadership, which primarily seeks to inspire followers through vision and charisma, authentic leadership promotes individualized support and value-driven interactions grounded in integrity and transparency. This makes it especially relevant in voluntary, non-elite sports environments where sustained engagement depends heavily on trust and relational quality (Agote et al., 2016).

Authentic leaders are thought to inspire trust and commitment by aligning their actions with deeply held values and demonstrating openness and integrity (Luthans and Avolio, 2003; Gardner et al., 2005). The four components of authentic leadership—self-awareness, relational transparency, internalized moral perspective, and balanced processing—are conceptually linked to interpersonal trust, which in turn influences self-efficacy, service quality perception, and intention to maintain relationships (Dirks and Ferrin, 2002; Bandura and Kavussanu, 2018; Walumbwa et al., 2008; Bandura, 2019; Baluku et al., 2020; Wei et al., 2018).

However, limited empirical research has examined these relationships in the context of recreational sports, particularly swimming programs. Prior studies have tended to focus on elite or youth athletes, leaving adult recreational participants underexplored. This study addresses this gap by proposing and testing a structural model that examines how swimming coaches’ authentic leadership affects participants’ trust, self-efficacy, perceived service quality, and relationship continuity intention. Specifically, it investigates whether coach trust acts as a mediator between authentic leadership and downstream outcomes. Based on theory, we anticipated that the effect of trust on relationship continuity intention would operate primarily through indirect pathways via self-efficacy and service quality, rather than as a strong direct effect.

**Figure 1:**
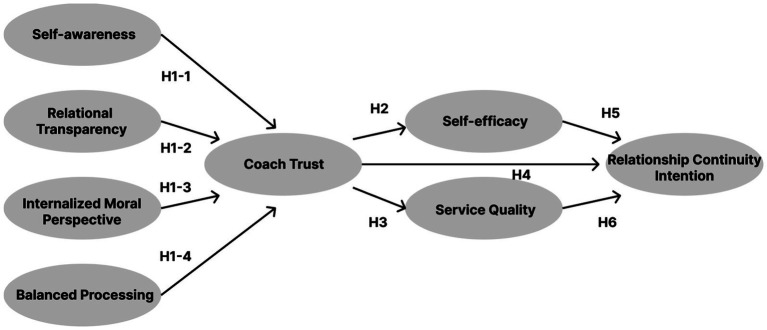
Research model.

The following hypotheses guide this study: (1) authentic leadership positively influences coach trust; (2) coach trust positively influences self-efficacy; (3) coach trust positively influences perceived service quality; (4) coach trust positively influences relationship continuity intention, primarily through indirect effects; (5) self-efficacy positively influences relationship continuity intention; and (6) service quality positively influences relationship continuity intention. The research model for hypothesis testing is shown in Figure 1.

## Methods

### Participants

Participants were adults enrolled in recreational swimming programs at local facilities across South Korea. A total of 300 individuals were recruited using convenience sampling, and after removing 20 incomplete or invalid responses, 280 valid questionnaires were retained for analysis. Eligibility criteria required participants to be 18 years or older and enrolled in a swimming program for at least 3 months, ensuring they had adequate experience to evaluate their coach and the program. The final sample consisted of 140 males (50%) and 140 females (50%), with a mean age of 35.6 years (SD = 9.8). Participants’ swimming experience ranged from 1 to 15 years (M = 5.3, SD = 4.1), indicating a heterogeneous group that included both novice and experienced swimmers. Table 1 presents detailed demographic characteristics of the participants.

**Table 1:** Demographic characteristics of participants.

Characteristic	Category	Frequency (*n*)	Percentage (%)
Gender	Male	80	36.9
	Female	137	63.1
Age Group	20–29 years	8	3.7
	30–39 years	47	21.6
	40–49 years	53	24.4
	50–59 years	60	27.7
	60 years and above	49	22.6
Swimming Experience	Less than 1 year	21	9.7
	Less than 5 years	68	31.3
	5 years of more	128	59.0
Occupation	Office worker	53	24.4
	Self-employed	15	6.9
	Homemaker	115	53.0
	Unemployed	34	15.7

### Measures

Validated scales from previous research were employed to measure the study constructs, using a 5-point Likert scale ranging from 1 (strongly disagree) to 5 (strongly agree).

**Authentic Leadership** was measured using the 16-item Authentic Leadership Questionnaire (ALQ) (Walumbwa et al., 2008), capturing four dimensions: self-awareness, relational transparency, internalized moral perspective, and balanced processing. During confirmatory factor analysis (CFA), two items (“My coach asks for feedback to improve” and “My coach admits mistakes when they occur”) were removed due to low standardized loadings (< 0.50) and high modification indices, suggesting redundancy. Their removal improved overall model fit and construct validity.

**Coach Trust** was assessed with a 7-item scale adapted from Dirks and Ferrin (2002).

**Self-Efficacy** was measured using a 6-item scale developed by Schwarzer et al. (2007), adapted for sport participants.

**Service Quality** was evaluated through a modified 10-item version of the SERVQUAL scale (Parasuraman et al., 1994), reflecting tangibility, reliability, responsiveness, assurance, and empathy within the swimming context.

**Relationship Continuity Intention** was assessed using a 5-item scale adapted from Berry and Parasuraman (1992).

The final number of retained items for each scale, along with their psychometric properties (e.g., AVE, CR), is reported in Table 2.

**Table 2:** Correlation analysis results.

Variable	Self-awareness	Relational transparency	Internalized moral perspective	Balanced processing	Coach trust	Self-efficacy	Service quality	Relationship continuity Intention
Self-Awareness	1							
Relational Transparency	0.622**	1						
Internalized Moral Perspective	0.640**	0.463**	1					
Balanced Processing	0.313**	0.173*	0.412**	1				
Coach Trust	0.453**	0.425**	0.453**	0.358**	1			
Self-Efficacy	0.424**	0.393**	0.510**	0.353**	0.519**	1		
Service Quality	0.596**	0.516**	0.619**	0.546**	0.649**	0.503**	1	
Relationship Continuity Intention	0.341**	0.337**	0.437**	0.424**	0.479**	0.671**	0.520**	1

**p < 0.01.

### Procedure

Data were collected over a one-month period in 2022 through both online and in-person surveys at swimming facilities nationwide. All participants were provided with an explanation of the study’s purpose and procedures, and informed consent was obtained in accordance with the guidelines of the Institutional Review Board of Kyungdong University (IRB No. 2022-004). Confidentiality and anonymity were maintained throughout the research process, and participation was voluntary.

### Data analysis

All analyses were conducted using SPSS 26.0 and AMOS 26.0. Preliminary analyses included descriptive statistics (means, standard deviations, frequencies) and normality checks using skewness, kurtosis, and Q-Q plots, confirming that parametric assumptions were met.

Internal consistency of the scales was verified using Cronbach’s alpha. Pearson’s correlation coefficients were calculated to examine interrelationships among the variables (see Table 3). Construct validity was tested using CFA. The main hypotheses were evaluated using structural equation modeling (SEM), with model fit assessed via standard indices: *χ*^2^/df (< 3.0), comparative fit index (CFI ≥ 0.90), Tucker-Lewis index (TLI ≥ 0.90), and root mean square error of approximation (RMSEA ≤ 0.08), following recommended thresholds (Hu and Bentler, 1999; Kline, 2023).

To evaluate indirect effects—particularly the mediating role of self-efficacy and service quality between coach trust and relationship continuity intention—a bootstrapping procedure with 5,000 resamples was performed.

## Results

### Descriptive statistics and reliability

Descriptive statistics and correlation coefficients among the study variables are presented in Tables 1, 2. The mean scores for each construct were generally high, suggesting favorable perceptions among participants. Internal consistency was strong across all scales, with Cronbach’s alpha coefficients exceeding 0.85: Authentic Leadership (M = 4.12, SD = 0.58, *α* = 0.91), Coach Trust (M = 4.25, SD = 0.63, *α* = 0.89), Self-Efficacy (M = 4.18, SD = 0.61, *α* = 0.87), Service Quality (M = 4.10, SD = 0.57, *α* = 0.88), and Relationship Continuity Intention (M = 4.22, SD = 0.60, *α* = 0.85). None of the reliability coefficients exceeded 0.95, indicating that item redundancy was not a concern (see Table 2).

### Measurement model

Confirmatory factor analysis (CFA) was conducted to examine the validity of the measurement model. As shown in Table 3, the model demonstrated good fit (*χ*^2^/df = 2.35, CFI = 0.96, TLI = 0.95, RMSEA = 0.056). All factor loadings exceeded 0.70 and were statistically significant (*p <* 0.001), supporting convergent validity. To further establish discriminant validity, the square root of the average variance extracted (AVE) for each construct was compared with the inter-construct correlations. As shown in Table 2, the AVE values exceeded the squared correlations among constructs, confirming discriminant validity.

**Table 3:** Confirmatory factor analysis (CFA) and reliability.

Factor	Items	SC	SE	*t*-value	*α*	CR	AVE
Self-awareness	The coach accurately understands their strengths and abilities.	0.707	0.405	-	0.807	0.841	0.570
	The coach seeks feedback to improve interpersonal relationships.	0.796	0.245	10.216			
	The coach knows when to express their opinions.	0.741	0.367	9.652			
	The coach understands the impact of their actions on others.	0.641	0.562	8.469			
Relational Transparency	The coach expresses their opinions clearly.	0.799	0.346	-	0.932	0.936	0.787
	The coach openly acknowledges their mistakes.	0.836	0.266	17.616			
	The coach encourages members to express honest opinions.	0.934	0.091	16.362			
	The coach is willing to speak difficult truths.	0.920	0.125	16.082			
Internalized Moral Perspective	The coach acts according to their beliefs.	0.843	0.242	–	0.908	0.919	0.741
	The coach sincerely expresses their emotions.	0.838	0.314	14.989			
	The coach emphasizes following thoughts and values.	0.870	0.227	15.875			
	The coach acts in accordance with their moral standards.	0.834	0.221	14.889			
Balanced Processing	The coach listens to opinions that oppose their own.*	–	–	–	0.873	0.806	0.583
	The coach pays attention to diverse opinions from members.	0.734	0.745	–			
	The coach reviews relevant information before making decisions.	0.892	0.372	12.637			
	The coach listens to opinions necessary for maintaining good relationships with members.	0.892	0.408	12.638			
Coach Trust	The coach makes efforts to treat members fairly.*	–	–	–	0.928	0.925	0.805
	The coach does not deceive members for personal gain.	0.895	0.286	–			
	The coach is honest with members.	0.920	0.156	18.749			
	I can trust the coach no matter the circumstances.	0.924	0.163	18.850			
Self-Efficacy	I can achieve the goals I have set for myself.	0.900	0.193	–	0.947	0.948	0.820
	I can overcome many challenges successfully.	0.941	0.116	23.156			
	I can perform well even in difficult situations.	0.922	0.157	21.960			
	I can successfully complete everything I need to do.	0.857	0.254	18.406			
Service Quality	The coach consistently helps members.*	–	–	–	0.910	0.927	0.809
	The coach communicates frequently with members.	0.894	0.159	–			
	The coach provides accurate services that meet the members’ needs.	0.907	0.139	19.251			
	The coach responds promptly to the members’ requests.	0.836	0.251	16.406			
Relationship Continuity Intention	I am willing to maintain a positive relationship with the coach.	0.908	0.122	–	0.907	0.923	0.752
	I will prioritize maintaining an ongoing relationship with the coach.	0.894	0.153	18.850			
	I intend to maintain a positive relationship with the coach in the future.	0.806	0.262	15.578			
	I am willing to continue taking swimming lessons from the coach.	0.710	0.381	12.523			

*χ*^2^ = 626.434, df = 347, p = 0.000, GFI = 0.841, CFI = 0.947, TLI = 0.938, RMR = 0.066, RMSEA = 0.061. *Items marked with an asterisk were removed during confirmatory factor analysis (CFA).

### Structural model

The hypothesized structural model was tested using SEM, and the results indicated a good model fit (*χ*^2^/df = 2.41, CFI = 0.95, TLI = 0.94, RMSEA = 0.059). Table 4 summarizes the results for each path:

**Table 4:** Hypothesis testing results.

Hypothesis	Path	Estimate	Standard error	*t-*value	*p*-value	Decision
H1-1	Self-Awareness → Coach Trust	0.31	0.117	2.642	0.008	Supported
H1-2	Relational Transparency → Coach Trust	0.169	0.067	2.522	0.012	Supported
H1-3	Internalized Moral Perspective → Coach Trust	0.179	0.068	2.642	0.008	Supported
H1-4	Balanced Processing → Coach Trust	0.225	0.05	4.512	0	Supported
H2	Coach Trust → Self-Efficacy	0.531	0.068	7.87	0	Supported
H3	Coach Trust → Service Quality	1.18	0.134	8.818	0	Supported
H4	Coach Trust → Relationship Continuity Intention	0.052	0.073	0.716	0.474	Not Supported
H5	Self-Efficacy → Relationship Continuity Intention	0.439	0.058	7.638	0	Supported
H6	Service Quality → Relationship Continuity Intention	0.226	0.074	3.036	0.002	Supported

*H1*: Authentic leadership had a significant positive effect on coach trust (*β* = 0.68, *p <* 0.001).

*H2*: Coach trust significantly predicted self-efficacy (*β* = 0.52, *p <* 0.001).

*H3*: Coach trust also significantly influenced perceived service quality (*β* = 0.47, *p <* 0.001).

*H4*: Contrary to expectations, coach trust did not have a direct effect on relationship continuity intention (*β* = 0.12, *p* = 0.128). This non-significant result represents an important divergence from prior literature, where trust has often been found to directly sustain relationship outcomes.

*H5*: Self-efficacy positively influenced relationship continuity intention (*β* = 0.45, *p <* 0.001).

*H6*: Service quality significantly predicted relationship continuity intention (*β* = 0.39, *p <* 0.001).

### Mediation analysis

To assess indirect effects, a bootstrapping procedure with 5,000 resamples was performed. The analysis revealed that coach trust significantly mediated the effects of authentic leadership on both self-efficacy (indirect effect = 0.35, 95% CI [0.24, 0.47], *p <* 0.01) and service quality (indirect effect = 0.32, 95% CI [0.20, 0.45], *p <* 0.01). In addition, self-efficacy (indirect effect = 0.23, 95% CI [0.14, 0.34], *p <* 0.01) and service quality (indirect effect = 0.18, 95% CI [0.09, 0.28], *p <* 0.01) both significantly mediated the relationship between coach trust and relationship continuity intention.

These findings suggest that while trust alone may not directly sustain participants’ long-term commitment, it indirectly promotes continuity through its positive effects on psychological (self-efficacy) and service-related (perceived quality) outcomes. This underscores the multi-step relational process through which authentic leadership impacts retention in recreational sports contexts.

## Discussion

This study explored the relationships among swimming coaches’ authentic leadership, coach trust, self-efficacy, service quality, and relationship continuity intention using structural equation modeling. Most of the hypothesized relationships were supported, offering both theoretical and practical insights into how leadership behaviors shape psychological and relational outcomes in community sports programs.

### Theoretical implications

The findings reinforce the central role of authentic leadership in fostering trust between coaches and participants, consistent with previous research (Walumbwa et al., 2011a,b). The four dimensions of authentic leadership—self-awareness, relational transparency, internalized moral perspective, and balanced processing—were all found to significantly contribute to participants’ trust in their coaches. This aligns with organizational psychology literature suggesting that authenticity in leadership enhances perceived integrity and promotes meaningful relationships (Avolio and Gardner, 2005).

Coach trust was also positively associated with self-efficacy and perceived service quality, supporting the notion that trust acts as a psychological bridge that facilitates motivation and favorable evaluations (Robert and Wilbanks, 2012). These findings suggest that in recreational sports contexts such as swimming, where participation is voluntary and non-competitive, the relational dimension of trust may be amplified. Swimming requires continuous engagement in structured training routines, and the inherent risks of water-based activities may heighten participants’ reliance on the coach’s credibility and integrity. Thus, trust may be even more central in swimming than in other recreational activities.

Notably, however, coach trust did not directly influence relationship continuity intention, contradicting several prior studies in service and sports contexts (Swan et al., 1999; Bateman and Valentine, 2015; Yagil and Medler-Liraz, 2013). This divergence may be attributable to cultural factors, as cross-cultural leadership research indicates that in collectivist societies, respect for authority figures does not always translate into sustained behavioral commitment (House et al., 2004; Rockstuhl et al., 2011). For adult recreational swimmers in South Korea, decisions to continue may depend more on individual efficacy or perceptions of program quality than on relational loyalty alone.

Importantly, the mediating roles of self-efficacy and service quality were confirmed, suggesting that trust operates indirectly, exerting its influence through more tangible or self-relevant mechanisms. This supports emerging sport psychology frameworks that emphasize multi-step motivational processes rather than direct relational outcomes. Future research might explore whether satisfaction, enjoyment, or perceived autonomy mediate similar relationships, particularly across different sports and cultural settings.

### Practical implications

The study highlights the importance of developing authentic leadership qualities in community sport coaches. Traits such as transparency, consistency, and moral integrity can significantly strengthen participant trust (Bandura and Kavussanu, 2018), which in turn enhances their perceived service experience and psychological engagement. To make this actionable, training programs for coaches should include structured reflective practice sessions that encourage coaches to examine their leadership values, and workshops on delivering constructive, individualized feedback. Incorporating tools such as peer observation, video-based self-reflection, and feedback logs can help coaches systematically build authenticity and trust in daily practice.

Moreover, the positive effects of coach trust on self-efficacy and service quality reinforce the need to prioritize trust-building as a strategic lever in program design and coach-participant interactions (Malloy and Kavussanu, 2021). Sports organizations should foster communication environments where mutual respect and personal attention are emphasized (Han et al., 2019). Ensuring program consistency, providing structured feedback opportunities, and cultivating a welcoming atmosphere can all contribute to sustained engagement.

Finally, the study underscores that fostering long-term commitment requires more than interpersonal trust alone. Coaches and program managers should focus on building participants’ confidence in their abilities (e.g., through individualized feedback and goal-setting strategies) and ensuring high-quality service provision (e.g., reliable instruction, clean facilities, adequate scheduling). These factors, more than trust in isolation, appear to be key drivers of continued participation in recreational swimming programs (Hashemi et al., 2024; Wang and Tseng, 2019).

Overall, these findings suggest that authentic leadership creates the relational and psychological conditions necessary for sustainable engagement—but only when paired with mechanisms that empower and satisfy participants.

### Limitation

#### Limitations and future research

While this study offers meaningful insights into the relational mechanisms linking authentic leadership, coach trust, self-efficacy, service quality, and relationship continuity intention, several limitations should be acknowledged to contextualize its findings and guide future research.

First, the study sample was limited to participants in recreational swimming programs in South Korea. Although this population provides valuable perspectives, the findings may not generalize to other sports, age groups, or cultural contexts. Cultural values related to authority, group identity, and interpersonal expectations may influence how participants interpret leadership behaviors or define relational continuity. Future studies should examine similar models in other countries and across diverse sporting environments, including both team-based and individual sports.

Second, the cross-sectional design restricts the ability to infer causal relationships among the variables. While structural equation modeling (SEM) provides robust estimates of associations, it does not establish directionality. To better understand the temporal and causal pathways linking authentic leadership to participant outcomes, longitudinal or experimental designs are recommended.

Third, the study relied exclusively on self-reported data, which may be subject to social desirability bias or inaccuracies in self-perception. For instance, participants might overstate their trust in coaches or underestimate negative experiences. Incorporating objective indicators—such as behavioral observations, retention records, or third-party assessments—would strengthen the validity of future studies.

Fourth, although this study confirmed the mediating roles of self-efficacy and service quality, it did not test potential moderating effects. Variables such as gender, age, or program duration may shape the strength or direction of the observed relationships. Future research should explicitly test moderator effects to identify the boundary conditions under which authentic leadership is most impactful.

Finally, the specificity of the swimming context may limit applicability to sports with different interaction structures or participant expectations. For instance, participants in team sports may place higher emphasis on peer dynamics or group cohesion than on individual trust in coaches. Comparative studies across multiple sports can help test the model’s robustness and refine its generalizability.

Addressing these limitations in future research will not only strengthen theoretical clarity but also offer practical insights for designing leadership interventions that foster lasting engagement and trust in diverse community sport settings.

## Conclusion

This study contributes to the growing body of literature on leadership in community sports by demonstrating the significant role of authentic leadership in fostering trust, enhancing self-efficacy, and improving perceptions of service quality among participants in recreational swimming programs. Importantly, the findings confirm that trust did not directly predict relationship continuity intention; rather, its influence operated indirectly through psychological (self-efficacy) and service-related (perceived quality) mechanisms. These indirect pathways highlight the complexity of relational dynamics in sustaining long-term engagement.

These results underscore the importance of developing leadership capacities in coaches, particularly those grounded in authenticity, ethical behavior, and interpersonal transparency. Sports organizations and training institutions should invest in leadership development programs that emphasize these qualities to improve both participant experiences and program retention.

Moreover, the study highlights the need for a more holistic approach to leadership research in sport—one that integrates personal, relational, and contextual factors. Future research should examine these dynamics across various sports, age groups, and cultural settings, employing longitudinal or experimental designs to uncover causal mechanisms and boundary conditions.

In sum, authentic leadership is a powerful driver of participant success and satisfaction in community sports. By fostering trust that indirectly sustains continuity, and by promoting psychological empowerment and service quality, it lays the foundation for more sustainable, inclusive, and participant-centered coaching environments.

## Data Availability

The raw data supporting the conclusions of this article will be made available by the authors, without undue reservation.

## References

[ref1] Agote L. Aramburu N. Lines R. (2016). Authentic leadership perception, trust in the leader, and followers’ emotions in organizational change processes. J. Appl. Behav. Sci. 52, 35–63. doi: 10.1177/0021886315617531

[ref2] Avolio B. J. Gardner W. L. (2005). Authentic leadership development: getting to the root of positive forms of leadership. Leadersh. Q. 16, 315–338. doi: 10.1016/j.leaqua.2005.03.001

[ref3] Baluku M. M. Matagi L. Otto K. (2020). Exploring the link between mentoring and intangible outcomes of entrepreneurship: the mediating role of self-efficacy and moderating effects of gender. Front. Psychol. 11:1556. doi: 10.3389/fpsyg.2020.01556, PMID: 32719643 PMC7347798

[ref4] Bandura C. T. (2019). An examination of authentic leadership in coaches and its consequenses for athletes (doctoral dissertation). Birmingham: University of Birmingham.

[ref5] Bandura C. T. Kavussanu M. (2018). Authentic leadership in sport: its relationship with athletes’ enjoyment and commitment and the mediating role of autonomy and trust. Int. J. Sports Sci. Coach. 13, 968–977. doi: 10.1177/1747954118768242

[ref6] Bateman C. Valentine S. (2015). The impact of salesperson customer orientation on the evaluation of a salesperson’s ethical treatment, trust in the salesperson, and intentions to purchase. J. Pers. Sell. Sales Manage. 35, 125–142. doi: 10.1080/08853134.2015.1010538

[ref7] Berry L. L. Parasuraman A. (1992). Prescription for a service quality revolution in America. Organ. Dyn. 20, 5–15.

[ref8] Dirks K. T. Ferrin D. L. (2002). Trust in leadership: Meta-analytic findings and implications for research and practice. J. Appl. Psychol. 87, 611–628. doi: 10.1037/0021-9010.87.4.611, PMID: 12184567

[ref9] Eime R. M. Harvey J. T. Charity M. J. Casey M. M. Westerbeek H. Payne W. R. (2016). Age profiles of sport participants. BMC Sports Sci. Med. Rehabil. 8:6. doi: 10.1186/s13102-016-0031-3, PMID: 26973792 PMC4788892

[ref10] Gardner W. L. Avolio B. J. Luthans F. May D. R. Walumbwa F. O. (2005). Can you see the real me?: a self-based model of authentic leader and follower development. Leadersh. Q. 16, 343–372. doi: 10.1016/j.leaqua.2005.03.003

[ref11] Han H. Yu J. Chua B. L. Lee S. Kim W. (2019). Impact of core-product and service-encounter quality, attitude, image, trust and love on repurchase: full-service vs low-cost carriers in South Korea. Int. J. Contemp. Hospit. Manag. 31, 1588–1608. doi: 10.1108/IJCHM-05-2018-0376

[ref12] Hashemi M. Farahbakhsh S. Geraei E. (2024). Investigating the effect of principals’ good character on secondary schools teachers’ self-efficacy with mediating role of coaching leadership style. J. Sch. Adm. 12:22. doi: 10.22034/JSA.2024.141394.2522

[ref13] House R. J. Hanges P. J. Javidan M. Dorfman P. W. Gupta V. (2004). Culture, leadership, and organizations: The GLOBE study of 62 societies. London: Sage publications.

[ref14] Howat G. Assaker G. (2013). The hierarchical effects of perceived quality on perceived value, satisfaction, and loyalty: empirical results from public, outdoor aquatic centres in Australia. Sport Manage. Rev. 16, 268–284. doi: 10.1016/j.smr.2012.10.001

[ref15] Hu L. T. Bentler P. M. (1999). Cutoff criteria for fit indexes in covariance structure analysis: conventional criteria versus new alternatives. Struct. Equ. Model. 6, 1–55. doi: 10.1080/10705519909540118

[ref16] Kline R. B. (2023). Principles and practice of structural equation modeling. New York, NY: Guilford publications.

[ref18] Lorimer R. Jowett S. (2014). The influence of role and gender in the empathic accuracy of coaches. Psychol. Sport Exerc. 15, 132–139. doi: 10.1016/j.psychsport.2013.10.004

[ref19] Luthans F. Avolio B. J. (2003). “Authentic leadership development” in Positive organizational scholarship. eds. Cameron K. S. Dutton J. E. Uinn R. E. (San Francisco, CA: Berrett-Koehler), 241–261.

[ref20] Malloy E. Kavussanu M. (2021). The effects of an authentic coaching intervention on athlete outcomes: a pilot randomised controlled trial. Psychol. Sport Exerc. 57:101957. doi: 10.1016/j.psychsport.2021.101957

[ref21] Ministry of Culture, Sports, and Tourism (2022). 2022 white paper on sports. Seoul: Ministry of Culture, Sports and Tourism (MCST).

[ref22] Parasuraman A. Berry L. L. Zeithaml V. A. (1994). Reassessment of expectations as a comparison standard and measuring service quality: implications for further research. J. Mark. 58, 111–124.

[ref23] Robert C. Wilbanks J. E. (2012). The wheel model of humor: humor events and affect in organizations. Hum. Relat. 65, 1071–1099. doi: 10.1177/0018726711433133

[ref24] Rockstuhl T. Seiler S. Ang S. Van Dyne L. Annen H. (2011). Beyond general intelligence (IQ) and emotional intelligence (EQ): the role of cultural intelligence (CQ) on cross-border leadership effectiveness in a globalized world. J. Soc. Issues 67, 825–840. doi: 10.1111/j.1540-4560.2011.01730.x

[ref25] Schwarzer R. Schüz B. Ziegelmann J. P. Lippke S. Luszczynska A. Scholz U. (2007). Adoption and maintenance of four health behaviors: Theory-guided longitudinal studies on dental flossing, seat belt use, dietary behavior, and physical activity. Annals of behavioral medicine, 33, 156–166. 17447868 10.1007/BF02879897

[ref26] Soto Garcia D. Garcia Herrero J. A. Carcedo R. J. Sanchez Garcia M. (2021). The impact of an authentic sports leadership program for coach. Front. Psychol. 12:701134. doi: 10.3389/fpsyg.2021.701134, PMID: 34248804 PMC8262451

[ref27] Swan J. E. Bowers M. R. Richardson L. D. (1999). Customer trust in the salesperson: an integrative review and meta-analysis of the empirical literature. J. Bus. Res. 44, 93–107.

[ref28] Vella S. A. Oades L. G. Crowe T. P. (2011). The role of the coach in facilitating positive youth development: moving from theory to practice. J. Appl. Sport Psychol. 23, 33–48. doi: 10.1080/10413200.2010.511423

[ref29] Walumbwa F. Avolio B. Gardner W. Wernsing S. Peterson T. (2008). Authentic leadership: development and validation of a theory-based measure. J. Manage. 34, 89–126. doi: 10.1177/01492063073089

[ref30] Walumbwa F. O. Christensen A. L. Hailey F. (2011a). Authentic leadership and the knowledge economy: sustaining motivation and trust among knowledge workers. Organ. Dyn. 40, 110–118. doi: 10.1016/j.orgdyn.2011.01.005

[ref31] Walumbwa F. O. Luthans F. Avey J. Oke A. (2011b). Authentically leading groups: the mediating role of positivity and trust. J. Organ. Behav. 32, 4–24. doi: 10.1002/job.653

[ref32] Wang C. J. Tseng K. J. (2019). Effects of selected positive resources on hospitality service quality: the mediating role of work engagement. Sustainability 11:2320. doi: 10.3390/su11082320

[ref33] Wei F. Li Y. Zhang Y. Liu S. (2018). The interactive effect of authentic leadership and leader competency on followers’ job performance: the mediating role of work engagement. J. Bus. Ethics 153, 763–773. doi: 10.1007/s10551-016-3379-0

[ref34] Yagil D. Medler-Liraz H. (2013). Moments of truth: examining transient authenticity and identity in service encounters. Acad. Manag. J. 56, 473–497. doi: 10.5465/amj.2011.0252

